# *In Vivo* Transcription Kinetics of a Synthetic Gene Uninvolved in Stress-Response Pathways in Stressed *Escherichia coli* Cells

**DOI:** 10.1371/journal.pone.0109005

**Published:** 2014-09-30

**Authors:** Anantha-Barathi Muthukrishnan, Antti Martikainen, Ramakanth Neeli-Venkata, Andre S. Ribeiro

**Affiliations:** Laboratory of Biosystem Dynamics, Department of Signal Processing, Tampere University of Technology, Tampere, Finland; University of Houston, United States of America

## Abstract

The fast adaptation of *Escherichia coli* to stressful environments includes the regulation of gene expression rates, mainly of transcription, by specific and global stress-response mechanisms. To study the effects of mechanisms acting on a global level, we observed with single molecule sensitivity the effects of mild acidic shift and oxidative stress on the *in vivo* transcription dynamics of a probe gene encoding an RNA target for MS2d-GFP, under the control of a synthetic promoter. After showing that this promoter is uninvolved in fast stress-response pathways, we compared its kinetics of transcript production under stress and in optimal conditions. We find that, following the application of either stress, the mean rates of transcription activation and of subsequent RNA production of the probe gene are reduced, particularly under oxidative stress. Meanwhile, the noise in RNA production decreases under oxidative stress, but not under acidic shift. From distributions of intervals between consecutive RNA productions, we infer that the number and duration of the rate-limiting steps in transcription initiation change, following the application of stress. These changes differ in the two stress conditions and are consistent with the changes in noise in RNA production. Overall, our measurements of the transcription initiation kinetics of the probe gene indicate that, following sub-lethal stresses, there are stress-specific changes in the dynamics of transcription initiation of the probe gene that affect its mean rate and noise of transcript production. Given the non-involvement of the probe gene in stress-response pathways, we suggest that these changes are caused by global response mechanisms of *E. coli* to stress.

## Introduction

Bacteria possess stress-response mechanisms [1]–[5] which, in response to sensing signals, orchestrate organized responses that lead to or include rapid changes in the cellular metabolism [1], [3], [5]–[9]. These responses have both specific and global effects on the gene expression profile [6], [10]–[14].

Studies using two-dimensional gels and transposable *lac* fusions [15] initially identified specific responses to stress conditions such as temperature shifts [16], pH shifts [17] DNA damage [18] and starvation [19]. In particular, it was identified that these conditions activate specific sets of genes. In general, the genes activated code for proteins governing the repair and recovery mechanisms and are responsible for altering the metabolic state [6], [20], [21].

Genome-wide microarray studies revealed changes in the transcriptomic profile, such as the up-regulation and down-regulation of specific groups of genes in response to stress inducers. For example, when subject to acidic conditions, genes encoding glutamate decarboxylase, *gadA* and *gadB* are significantly induced, which provides acid resistance via consumption of protons leaking into cells during extreme acidic conditions [22], [23]. Another study [14] distinguished three classes of changes in gene expression following rapid acid treatment. These are up-regulation with recovery (e.g. *fimB*, *ygaC*, and *yodA*), up-regulation without recovery (*hdeB*, *glpF*, and *hdeA*) and, delayed response to acid (e.g., *nuo* and *hsl*). The delayed acid response was hypothesized to be a secondary effect of an acid-associated metabolism, rather than a direct response to cytoplasmic acidification [14].

Similarly, being a facultative anaerobe, *E. coli* also has the means to deal with oxidative stress [24]–[27]. The adaptive response to reactive oxygen species (ROS) exposure, such as super oxide and peroxide is triggered by regulons, namely, *SoxRS* and *OxyR* [28]–[30]. The *SoxRS* regulon prevents cellular damage by superoxide and nitric oxide by activating the superoxide dismutase system [13]. Meanwhile, the *OxyR* regulon protects cells from damage by peroxides such as Hydrogen peroxide (H_2_O_2_) by coding for a transcriptional activator, OxyR, which activates genes encoding catalases (*katG*), alkyl hydrogen peroxide reductases (*ahpCF*), and small regulatory RNA (*OxyS*) [31], [32].

Aside the triggering of specific responses, there are also global changes in the expression level of genes that, while unrelated to stress-response regulators or effectors [6], [7], [22], [33], are instead involved in metabolism regulation, membrane transport, transcription and translation, among others [9], [34]. These global changes are believed to balance energy-efficient growth with the costs of increased activity of stress-related genes [7], aiding survival at reduced growth rates [4], [5], [12]. Some of these global changes are believed to be implemented by alternative sigma (σ) factors [4], [10], [11], [33], [35] and small nucleotides accumulated in stress conditions, namely, guanosine pentaphosphate or tetraphosphate (p) ppGpp [6], [36], [37], among others.

Gene expression dynamics in *E. coli* are believed to be mainly controlled at the stage of transcription, particularly its initiation [38], since subsequent stages, namely elongation and termination, as well as translation elongation, are much faster [39]–[42] and only rarely are halted or prematurely terminated [43], [44]. Transcription initiation is a multi-stepped process [45]. *In vitro* [45]–[47] and recent *in vivo* studies [48]–[52] showed that, under optimal conditions for the promoters studied, at least two of the steps in transcription initiation limit the rate of RNA production. Namely, their dynamics of RNA production is well fit by a sub-Poissonian model of two or more steps, each exponentially distributed in duration [48]–[52].

The RNA production kinetics is regulated by molecules that affect the duration of these steps [45], [50], [53]. The duration of these steps is also influenced by environmental factors [52], [54], is sensitive to small changes in the promoter sequence [54], and varies from one event to the next [48]–[52]. The latter is a strong source of noise in RNA production [48]–[52].

Previous studies using MS2d-GFP tagging of RNA showed that, in stable environmental conditions, once the target gene is activated and if the inducer concentration is kept constant, the mean rate of transcript production does not change significantly over time [48], [51], [52]. Similar results were obtained using microarrays, at a genome wide level [34]. Meanwhile, stress-response mechanisms alter the cellular metabolism [7], [9], [34]. It has been suggested that such changes include a reduction in the expression rate of many non-stress-related genes [34].

Here, we investigate how mild acidic shift [17], [22] and oxidative stress [9], [24], [25] affect the *in vivo* transcriptional kinetics of a probe gene under the control of a synthetic promoter (P*_lac/ara-1_*) uninvolved in stress-response pathways. The probe gene codes for an RNA target for MS2d-GFP proteins [55], which allows its detection with single-molecule sensitivity, as soon as it is produced [55]. From the analysis of when new, individual RNA molecules from the probe gene appear in the cells under stress and in optimal conditions, we address the following questions. Does stress affect the kinetics of activation of the probe gene? Does stress affect the kinetics of transcription of the probe gene following activation? Are these changes immediate or gradual? Do the effects of the two stress conditions tested differ from one another? What changes in the kinetics of the intermediate stages of transcription are responsible for the observed changes in the kinetics of RNA production from the probe gene?

## Results

Our aim is to assess the effects of “mild” stress conditions on the dynamics of transcription of a gene uninvolved in stress-response pathways. By “mild”, we imply “cells with significantly reduced, but not null, division rates”. For this, we selected oxidative stress and acidic shift as the stress conditions since, first, it is possible to tune the degree of stress so as to attain the ‘mild stress’ conditions. Also, the effects of both stress conditions can be observed in reasonable short time scales [32]. Finally, it is possible, using a peristaltic pump for constant media refreshment, to maintain mild-stress conditions stable during the live cell imaging.

As described in the Methods section, microscopy measurements were made on cells in the exponential phase (OD_600_ of 0.45). The mild stress conditions were applied only at this stage of cell growth and after placing the cells under the microscope (see Methods section). Then, we followed, *in vivo* and with single-molecule sensitivity, the kinetics of transcript production of a synthetic gene on a single copy F plasmid. This gene, under the control of the *lac-ara1* promoter [46], encodes an RNA with an *mRFP1* coding region followed by 96 binding sites for MS2d-GFP reporter proteins [55]. These are coded from a medium-copy plasmid and, by binding to the target RNA molecules as soon as these are produced, allow their quick detection.

Images of cells were taken immediately after the application of stress, once per minute, for 4 hours (under dark conditions to prevent photolytic activities), allowing quick detection of both produced RNA molecules as well as of cell division events. In general, we performed two microscopy sessions per condition, since preliminary assessments showed no significant differences between sessions and because, in each session, several independent panels can be captured, so as to maximize the number of cells observed. No significant differences were found between sessions performed in the same conditions.

### Effects of the mild-stress conditions on cell division rates

To quantify the degree of stress on the cells during the measurements, we measured their division time from the images [7], [9], [34]. For that, we selected cells that were born from a cell present at the start of the measurements. In the rare cases that these cells of generation 1 (G1) did not divide until the end of the measurements, we assumed that they divided at that moment. For comparison, we obtained the same data from cells under optimal growth conditions (control). In total, we extracted this data from 477 cells in the control measurements, from 182 cells under acidic shift, and from 251 cells under oxidative stress.

The mean division time was found to be 66.7 min in control cells (optimal conditions), 87.2 min in cells under acidic shift, and 91.0 min in cells under oxidative stress, while the standard deviation of these times was found to be 38.4 min in control cells, 52.5 min in acidic shift and 70.3 min in oxidative stress. These numbers indicate that cells were stressed in the two latter conditions [7], [9], [34]. To confirm this, we performed Kolmogorov-Smirnov (KS) tests to determine whether the distributions of division times in both stress conditions could not be statistically distinguished from the distribution obtained from control cells. In both comparisons, the p-value was smaller than 10^−7^ and, thus, we conclude that the division times differed significantly from the control (usually, for p-values smaller than 0.01 it is accepted that two distributions differ significantly). We conclude that the cells under acidic shift and oxidative stress by constant media refreshment during the microscopy measurements were mildly stressed during that period of time, similarly to when in liquid culture.

### Effects of stress on the dynamics of transcription of the target gene when uninduced

Next, we verified if our target gene was, in some way, directly activated or repressed by any stress-response mechanism of *E. coli* to acidic shift or oxidative stress. For that, without inducing the target gene and 1 h after applying stress and inducing the plasmids coding for MS2d-GFP, we measured the number of target RNA molecules in individual cells from fluorescent microscopy images. For comparison, we also observed cells under optimal growth conditions (control). The mean number of tagged RNAs per cell equalled 0.14 in optimal, control conditions (206 cells), 0.10 in acidic shift (83 cells), and 0.16 in oxidative stress (216 cells). These mean numbers are, in all cases, much lower than in cells where the target gene is fully induced (∼3 RNA/cell 1 h after induction, in optimal conditions [48]). Further, the distributions of RNA numbers in individual cells in each of the three conditions could not be statistically distinguished from one another (p-values of the three, pairwise KS tests larger than 0.01). We conclude that neither the promoter controlling the production of the RNA target for MS2d-GFP is activated or repressed by any of the response mechanisms to acidic shift or to oxidative stress, neither is the detection mechanism of target RNA molecules affected. As such, this synthetic gene (here onwards referred to as ‘probe’ gene) can be used as a probe gene to study the kinetics of transcriptional dynamics of a promoter not belonging to stress-response pathways. As a side note, from this, it is also possible to conclude that neither stress condition affects the kinetics of binding of MS2-GFP molecules to the target RNA coded by the probe gene (e.g. by reducing the binding affinity) or the detection mechanism in any other significant way.

### Effects of stress on the kinetics of activation of the target gene

Previous studies have reported that stress leads to multiple changes in the cells, including a decrease in fluidity (and, thus, permeability) of the cell membranes that, in general, delay the transcriptional response to external signals [21], [56]–[58]. Therefore, from the time lapse microscopy measurements, we first studied how acidic shift and oxidative stress affect the activation time of our target gene in individual cells, following the introduction of the inducer, IPTG, in the media of cells under microscopy observation.

**Figure 1:**
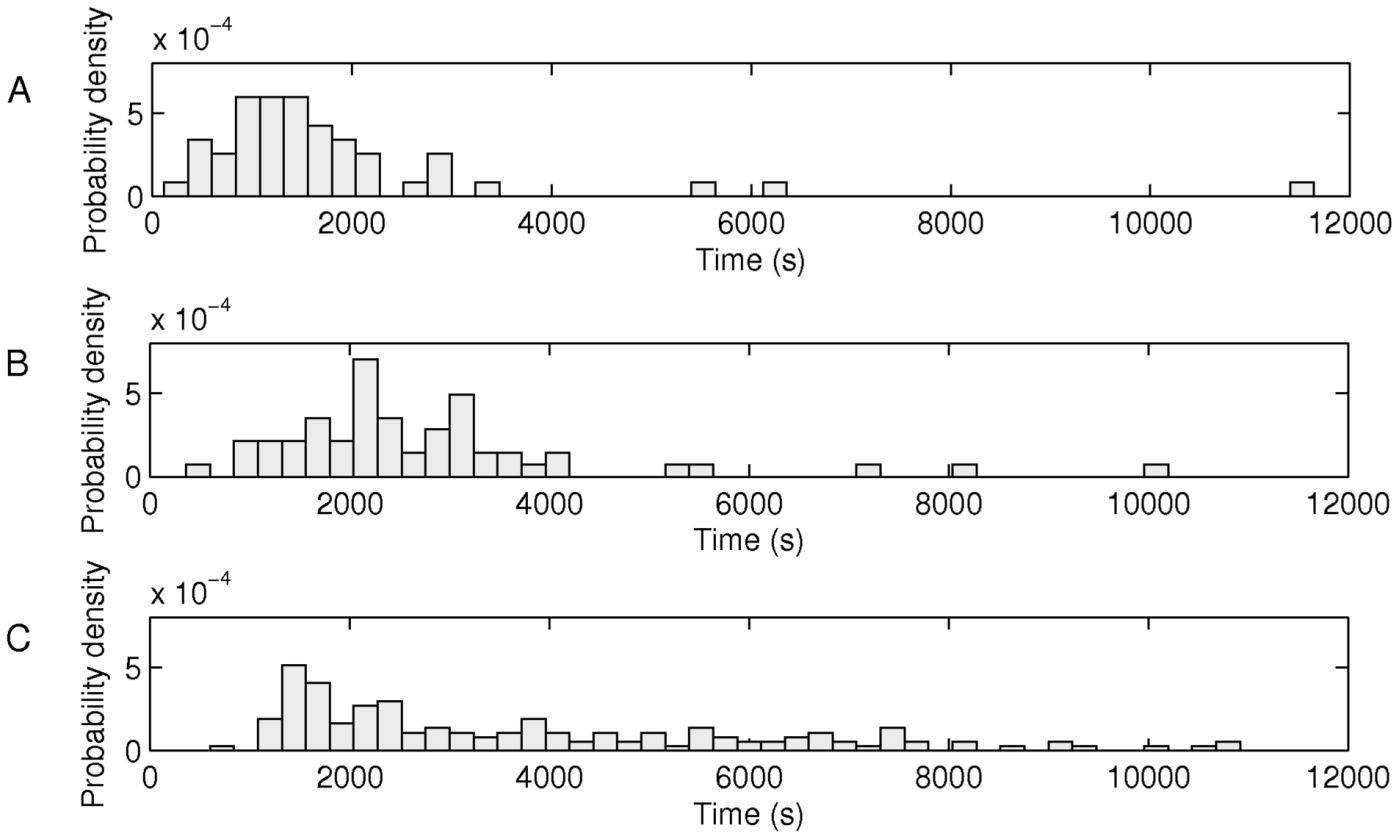
Transcription activation. Probability density distributions of measured activation times, t_0_, in individual cells subject to (A) optimal growth conditions (55 cells), (B) acidic shift (61 cells), and, (C) oxidative stress (158 cells). Mean and standard deviation of the distributions equalled, respectively, (A) μ(t_0_) = 1871 s and σ(t_0_) = 1819 s, (B) μ(t_0_) = 2960 s and σ(t_0_) = 2217 s, and (C) μ(t_0_) = 3931 s and σ(t_0_) = 2678 s.

From cells present at the start of the measurements, we extracted the time for the first target RNA to appear in each cell (named ‘activation time’, t_0_), in each stress condition and in control conditions, for comparison. Note that t_0_ includes both the time for a cell to uptake at least one inducer as well as the time for the first transcription event to be completed [51]. The distributions of values of t_0_ in each stress and the control are shown in Figure 1 (in the legend are given the mean and the standard deviation of each distribution, as well as the number of samples in each condition). Visibly, activation times are much longer in both stress conditions, particularly oxidative stress.

To determine whether the distributions in Figure 1 differed statistically from one another, we followed the same procedure as in [51]. Namely, we performed KS tests to compare all pairs of distributions of values of t_0_. We obtained p-values of 2.2×10^−8^, 4.3×10^−6^, and 0.0012, for the pairs of conditions control vs. oxidative, control vs. acidic shift, and oxidative vs. acidic shift, respectively. Since all p-values are much smaller than 0.01, we conclude that the activation time of the probe gene differs significantly between all conditions, in a statistical sense. In particular, this activation time is shorter in optimal, control conditions when compared to either stress condition. Also, it is significantly longer under oxidative stress than under acidic shift. The cell-to-cell variability in activation times, as measured by the squared coefficient of variation, CV^2^, also differs between the conditions. In particular, it equals 0.94 in control cells, 0.56 under acidic shift, and 0.46 in oxidative stress. Overall, these results provide strong evidence that the kinetics of intake of the inducer, IPTG, are significantly altered in both stress conditions.

### Effects of stress on the kinetics of transcription of the target gene

Next, we studied the temporal evolution of the kinetics of RNA production from the probe gene during the microscopy measurements, under both stress conditions. In each case, given the time scale of the duration of intervals between consecutive RNA productions and the duration of the measurements, we compare the mean and standard deviations of the intervals between consecutive RNA productions (in individual cells) that were initiated in the first hour of the measurements with those initiated during the second hour (Table 1). From Table 1, in both stress conditions, the mean duration of the intervals initiated in the second hour is longer than of those initiated during the first hour. In particular, in cells under acidic shift, they become 41% longer while in cells under oxidative stress they become 29% longer. Meanwhile, under oxidative stress, the variability of the intervals' duration (as measured by the CV^2^) also differs between intervals initiated during the first and the second hour, being much smaller in the latter ones. In cells under acidic shift, this quantity does not differ significantly between the two distributions of intervals. To test whether these changes in the kinetics of RNA production between the first and the second hour following the application of stress are statistically significant, we performed KS tests. We compared, for each stress condition, the distributions of interval initiated in the first and in the second hour. In both stress conditions, the p-value of the KS test was much smaller than 0.01, from which we conclude that the distributions differ, in a statistical sense. Thus, we conclude that, following the application of the stresses, there is a gradual reduction in the rate of transcription.

**Table 1:** Intervals between consecutive RNA productions in individual cells.

	Acidic Shift		Oxidative Stress	
	First hour	Second hour	First hour	Second hour
No. of cells at the start	95	135	352	463
No. of cells at the end	135	167	463	507
No. of intervals	87	94	116	178
μ_Δt_ (s)	866	1452	1351	1904
σ_Δt_ (s)	599	1054	1061	1063
σ_Δt_^2^/μ_Δt_^2^	0.48	0.53	0.62	0.31

Number of cells analysed from start to end of the measurement period, number of intervals between productions of consecutive RNA molecules in individual cells collected, mean duration (μ_Δt_) and standard deviation (σ_Δt_) of the intervals' durations for the first and for the second hour, and squared coefficient of variation (σ_Δt_^2^/μ_Δt_^2^) of these intervals' duration.

For comparison, we also performed the same analysis in cells under optimal control conditions. In these, in agreement with previous studies [48], [52], no significant differences were observed between the distributions of intervals initiated in the first and in the second hour of the measurements (p-value of the KS test equalled 0.02).

Finally, we compared the kinetics of RNA production under stress (see Table 1) and under optimal, control conditions. In control conditions, the mean of the intervals equalled 898 s and the CV^2^ equalled 0.60, throughout the two hours of measurements. As such, for cells under acidic shift, only the distribution of intervals initiated during the second hour of the measurements differed from the control (p-value<0.01 for intervals initiated in the second hour, and p-value of 0.82 for intervals initiated in the first hour). Meanwhile, in cells under oxidative stress, the changes in the kinetics of RNA production with stress appear to occur faster, since both the intervals initiated in the first hour and the intervals initiated in the second hour differed from the control (both p-values<0.01).

Given the difference in the values of the CV^2^ of the duration of the intervals initiated in the first hour and of the intervals initiated in the second hour of the measurements, one expects the cell to cell diversity in numbers of RNA molecules produced during those two periods of time to differ significantly. To assess this, for each condition, we obtained the number of RNA molecules in each cell from the total spot intensity distribution from all cells [59] at the end of the first hour of the measurements and at the end of the second hour of the measurements. From this, we found that under acidic shift this quantity equalled ∼1.7 at both time moments. However, under oxidative stress, it equalled ∼1.8 at the end of the first hour, and ∼1.2 at the end of the second hour. We conclude that the decrease over time in the variability of the duration of intervals between consecutive RNA molecules in cells under oxidative stress significantly affects the cell to cell diversity in RNA numbers.

It is of interest to note that, while RNA production is a sub-Poissonian process (CV^2^ of the intervals smaller than 1), the Fano factors of the numbers of RNA molecules are larger than 1. This is due to the dependence of the latter quantity, and only the latter quantity, on the variability between cells' lifetime and errors in partitioning of the tagged RNA molecules in cell division [60], among other factors.

**Figure 2:**
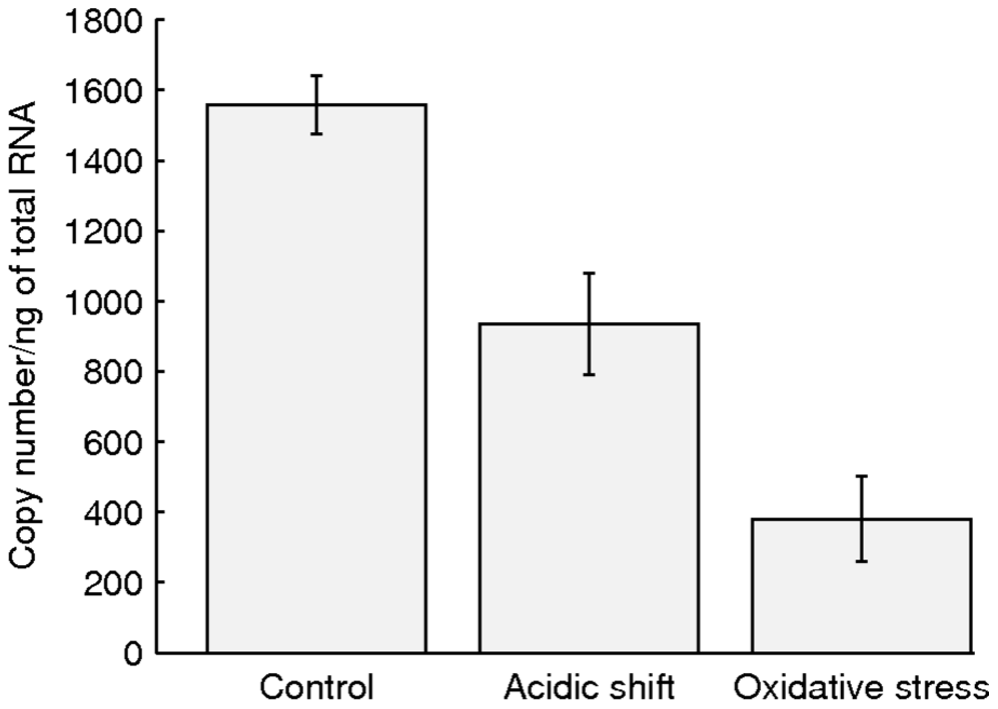
Quantification of the target gene mRNA copy number using an external calibration method. Error bars indicate the 95% confidence intervals of the mean of three separately calibrated experiments for each condition, assuming that the measurement error is Gaussian-like noise.

Finally, in comparison to the control, the mean duration of the intervals between productions of consecutive RNAs by the probe gene increased by 62% under acidic shift and by 112% under oxidative stress (log10 ratios of 0.2 and 0.32, respectively). This is in close agreement with the RNA expression ratios reported in [22] for non-stress-related genes. It is possible to confirm these differences by measuring absolute mRNA numbers by qPCR in both stress conditions (2 hours after stress application) and the control. The results (Figure 2) confirm that the transcription rate is higher in the control (shorter intervals between consecutive RNA molecules), followed by acidic shift, followed by oxidative stress, in agreement with the results in Table 1 extracted from the microscopy measurements. In particular, from the qPCR, the rate of target RNA production under acidic shift is 40% weaker than the control, while under oxidative stress it is 76% weaker.

### Effects of stress on the rate limiting steps in transcription of the target gene

As noted, the kinetics of RNA production remained sub-Poissonian throughout the course of the measurements in both stress conditions. Thus, similar to when under optimal or sub-optimal conditions [48]–[52], [61], transcription initiation of the probe gene under the control of the P*_lac/ara-1_*, in cells under mild stress can be well represented by a multiple rate-limiting step model (see [45]), with each step following an exponential distribution in duration [48]:
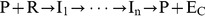
(1)where *P* is a promoter, *R* is the RNA polymerase, *I_i = 1,…,n_* is the promoter-RNA polymerase complex at different stages of initiation, and *E_C_* is the elongation complex. Assuming this model, it is possible to extract from the distribution of intervals between consecutive RNA productions the number and expected duration of the rate-limiting steps in transcription [62]. Previous studies of the kinetics of transcription under optimal growth conditions showed that, in these, usually there are two major rate-limiting steps [48]–[52], in agreement with results using *in vitro* techniques [54]. This agreement led to suggesting that the two observed steps in the in vivo measurements ought to be the closed and open complex formations that were reported from the *in vitro* measurements [48].

However, from Table 1, under oxidative stress, the CV^2^ of intervals starting at the second hour of the measurements is much smaller than 0.5. If the duration of each rate-limiting step is exponentially distributed, the CV^2^ of the intervals between consecutive RNA molecules can only be lower than 0.5, if there are more than 2 rate-limiting steps. This suggests that, in this stress condition alone, the number of significantly rate-limiting steps is higher than in the control (where the CV^2^ equals 0.6).

**Figure 3:**
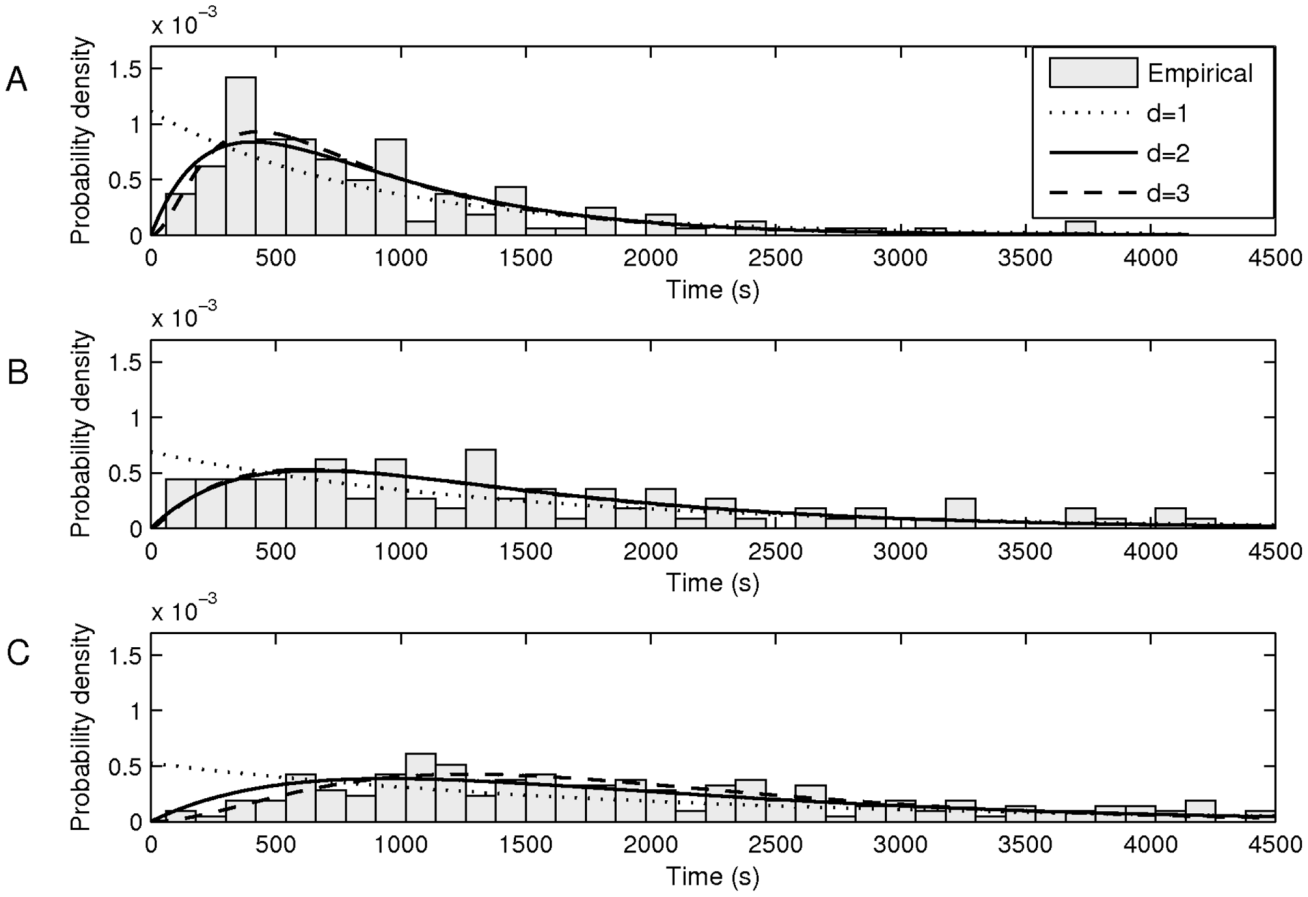
Probability density distributions of time intervals between productions of consecutive RNA molecules in individual cells in three conditions in the second hour following the application of stress conditions. (A) Control, optimal growth conditions, obtained from 139 cells (mean interval of 898 s and CV^2^ of 0.60). (B) Acidic shift, obtained from 167 cells (mean interval of 1452 s and CV^2^ of 0.53, and (C) Oxidative stress, obtained from 507 cells (mean interval of 1904 s and CV^2^ of 0.31). The probability density functions of best-fit inferred models with one (dotted line), two (solid line) and three (dashed line) exponentially-distributed rate-limiting steps are also shown (see Table 2). In some conditions, the 2-step and 3-step models cannot be visually distinguished.

To assess this, we studied the distributions of intervals between consecutive RNA productions that were initiated during the second hour of the measurements (Figure 3). As in [48], [50]–[52], from the distributions, we estimated by maximum likelihood (see Methods) the number and duration of the rate-limiting steps in transcription. Results are shown in both Figure 3 (see legend) and in Table 2 (log-likelihoods and steps' durations of the models) for each condition when assuming one, two, three, and four rate-limiting steps (results of the 4-step model are only shown in Table 2, as in no condition could they be distinguished visually or in a statistical sense from the 3-step model).

**Table 2:** Log-likelihood and durations of the steps of the inferred models with *d* steps, for each condition.

	Control		Acidic Shift		Oxidative Stress	
d	Log-likelihood	Duration (s)	Log-likelihood	Duration (s)	Log-likelihood	Duration (s)
1	−1052.99		−778.35		−1522.22	
2	−1035.75	(284, 614)	−770.34	(456, 996)	−1486.53	
3	−1033.96		−770.29		−1481.07	(635,635,635)
4	−1033.96		−770.28		−1481.07	

Log-likelihood of the inferred models with d steps, for each condition, from measurements during the second hour of the time-lapse microscopy. Also shown are the durations of the rate-limiting steps of the preferred model (the temporal order of the steps is unknown). Note that the log-likelihood does not increase for higher values of *d*, beyond the preferred models.

Table 2 shows the log-likelihood values for each d-step models, for each condition. Also shown are the durations of the inferred steps of the preferred model. The preferred model (i.e. number of rate-limiting steps) was determined by a likelihood-ratio test between pairs of models to reject a lower-degree model in favour of a higher-degree one [48]. Table 3 shows the results of these tests, for each condition. In the control and acidic shift conditions, a 2-step model is preferred, while in the case of oxidative stress, a 3-step model is preferred.

**Table 3:** Likelihood-ratio test P values between pairs of models for each condition.

d_0_, d_1_	Control	Acidic shift	Oxidative stress
(1, 2)	0.000	0.000	0.000
(2, 3)	0.059	0.771	0.001
(3, 4)	0.999	0.850	0.951

P values from the Likelihood-ratio test of comparison between pairs of models for each condition. Data from intervals starting during the second hour of the time-lapse microscopy. The null model is the d_0_ step model (where d_0_ is 1, 2, or 3) while the alternative model is the d_1_ step model (where d_1_ = d_0_+1). For P values above 0.01, the simplest model (i.e. lesser steps) is preferred.

Next, since the inference method can only provide the model that best fits the data, out of the set of models allowed (all steps need to be exponentially distributed in duration) we tested the accuracy with which the preferred, inferred models match the measurements. For that, we performed a KS test for each condition between the empirical cumulative distribution function and the corresponding cumulative distribution function of the preferred model. The resulting p-values were all larger than 0.01 (0.2 for control, 0.7 for acidic shift, and 0.6 for oxidative stress), from which we conclude that all models match the corresponding empirical data in a statistical sense.

From the inferred models (Table 3) we find that, compared to the control, the mean rate of transcription initiation in cells under acidic shift was reduced via an increase of the duration of the two rate-limiting steps. These increases are such that the ratio between them remained similar to the ratio in the control (one step is approximately double the length of the other), which can explain why the noise in transcription in this stress condition did not differ significantly from the control. On the other hand, according to the model of transcription inferred for cells under oxidative stress, the reduction in the transcript rate was achieved not only by increasing the duration of the two steps but also by an additional step in transcription becoming rate-limiting. Note that, provided that transcription is a multi-step process [45]–[48], [50], [52], [54], [67] and that each step follows an exponential distribution in duration, values of CV^2^<0.5 are possible only if there are at least three rate-limiting steps (as confirmed by the inference procedure).

Relevantly, it is noted that the rate-limiting steps detected likely occur in transcription initiation rather than elongation, since elongation only lasts for tens of seconds [43], [68] while the changes in the intervals between consecutive RNAs were of the order of hundreds of seconds. Further, events in elongation (e.g. transcriptional pauses) will contribute to the variability of the intervals, but not to their mean duration.

Finally, as a side note, the preferred 3-step model for cells under oxidative stress is such that all the steps appear to be of equal duration. As explained in the methods section, this is due to an unknown artefact that favours solutions where the intermediate steps are of identical size, when the steps are of similar duration. We performed a rough estimation (see methods) based on the number of samples of the minimum ratio between these durations that would allow the inference algorithm to be more likely to return a non-gamma solution. This ratio equalled 1.30. Thus, using this method of inference, we can only conclude that the three rate-limiting steps differ in duration by less than 30% in this case.

## Discussion

From the moments of appearance of the first target RNA and the subsequent time intervals between consecutive target RNA productions in individual, live cells, we studied the kinetics of transcription of a synthetic probe gene, following its induction, in *E. coli* cells subject to sub-lethal stress conditions, acidic shift and oxidative stress. In particular, we studied how this kinetics changes over time due to the metabolomic and transcriptomic changes that cells undergo under the mild stress conditions. To control the production of the target RNA, we used the *lac/ara-1* promoter, which we verified to be not directly affected by the stress-response mechanisms to the two stress conditions studied here.

First, we observed that the activation of the probe gene by external induction (as measured by the appearance of the first target RNA) was slower in stressed cells. The duration of this delay was found to be stress-dependent. This observation is in agreement with previous observations of genome-wide delays in transcriptional responses to external signals in cells subject to stress conditions [21], [56]–[58]. Notably, the activation time measured here includes both the time for a cell to intake inducers from the medium as well as the time to produce the first RNA, once the promoter is activated by the inducers [51]. Since the kinetics of transcription of activated genes did not differ widely from the control during the first hour following the application of stress (e.g. under acidic shift, no changes were detected), the main cause for the increase in the time for the appearance of the first RNA in the cells is likely a decreased rate of intake of inducers (IPTG), via diffusion through the membrane [63], by the stressed cells. This would be in agreement with the known reduction of membrane fluidity due to the down-regulation of OMP proteins under stress [21], [23], [56]–[58]. Finally, since the increase in the time for the first RNA to appear in the cells differed in the two stress conditions tested, it is reasonable to hypothesize that *E. coli* is able to fine-tune this decrease in fluidity of IPTG. Future studies should provide more information.

Next, we compared the distributions of intervals between consecutive transcription events in individual cells when under stress and when in optimal conditions. As part of the global response to stress, we observed a gradual, significant reduction of activity of the activated probe gene in stressed cells, in agreement with previous studies [21], [56], [58]. Both the degree and speed of this reduction, confirmed by qPCR, are stress-dependent. Under oxidative stress, the kinetics of transcription changed rapidly. Under acidic shift, the kinetics only differed significantly from the control in the second hour. This is, perhaps, not surprising, given the acid tolerance system in enteric bacteria [20], [64], [65]. Overall, the changes in the mean rate of RNA production (up to 40%) and in the degree of noise (up to 100%, as measured by the CV^2^) ought to be of significance to the cells' phenotype, as they are expected to affect significantly both the mean and variability of protein numbers. As a side note, these results support the hypothesis that the genome-wide reduction in RNA and protein numbers of genes unrelated to stress [21], [56], [57] is achieved by *E. coli* by decreasing transcription rates (suggested in [22], [34]) rather then, e.g. increasing the rates of RNA and/or protein degradation (which would be more energy-consuming).

Also as a side note, we did not assess whether the copy number of the single-copy F plasmid coding for the probe RNA was affected by the stress conditions. The observed reduction of activity suggests that the copy numbers did not increase. Also, there is no evidence of plasmid loss, as the activity reduction observed is not based on a halting of transcription in some cells. Nevertheless, such plasmid-loss would not have affected the conclusions, as these are based on the time for the first RNA to be produced and on the duration of intervals between consecutive RNAs in each cell.

Notably, the graduality of the stress responses in the transcription kinetics of the probe gene, supports the hypothesis that they are indirect consequences of the global response mechanisms to stress of *E. coli*. If, instead, the observed changes were due to changes in the physical parameters of the probe system (e.g. in the binding affinity of the MS2d-GFP proteins to the target RNA), or of the cell cytoplasm or membrane, they should have occurred rapidly. That is, its effects should be visible in the first hour following the application of stress, and remain stable thereafter. Instead, we expect the observed gradual changes to be a result of a changing cellular physiology due to the stress-response mechanisms, which take at least 30 to 60 minutes to occur, depending on the severity of the stress conditions [9], [66].

Finally, our results showed that the changes with stress on the dynamics of RNA production of the probe gene occurred at the level of the number and duration of the rate-limiting steps in transcription initiation, which allows tuning both rate and noise level in RNA production. Importantly, the changes were found to be stress-dependent.

Interestingly, while a change in the steps' duration, particularly of the closed complex formation, can be explained by, e.g., a decrease in the number of available RNA polymerases, changes in the kinetics and number of subsequent steps (e.g. open complex formation and promoter escape) require changes in the kinetics of interaction between RNA polymerase and promoter region of the target gene. Because of this, we hypothesize that the changes observed, in particular the increase in the number of rate-limiting steps under oxidative stress, were caused by changes in the populations of molecules component of, or regulators of, the RNA polymerase. We hypothesize that changes in the populations of σ factors [4], [10], [11], [33], [35] or ppGpp and pppGpp molecules [36], [37], [69] are the most likely explanations. Future studies are needed to assess this. Aside this, it would also be of interest to study the degree of changes in transcription dynamics to different degrees of stress and what occurs when cells are subject once again to optimal conditions.

## Conclusions

We used live, single-RNA detection techniques to investigate how mild acidic shift and oxidative stress affect the *in vivo* transcriptional kinetics of a synthetic promoter (P*_lac/ara-1_*) that is uninvolved in the stress-response pathways of *E. coli*.

From the activation time of the probe gene in individual cells under optimal conditions and under each of the two stress conditions, it is possible to conclude that, in general, stress increases the mean activation time of the probe gene. This increase is stress-dependent. Interestingly, the cell-to-cell variability in activation times decreased significantly in stressed cells, from which we conclude that the effects of stress on the cells' intake processes were fairly homogenous.

The changes in the distributions of intervals between consecutive RNA productions following the application of stress allow concluding that, in stressed cells, the rate of transcript production by the probe gene decreases gradually. From the analysis of these distributions of intervals it was further possible to infer how the RNA production rates were reduced. While under acidic shift this reduction was achieved by an increase in duration of the same two rate-limiting steps in transcription observed under optimal conditions, under oxidative stress a third rate limiting step emerged, explaining how it was possible to achieve a strong reduction of the noise in RNA production as well.

Overall, we conclude that, following the application of stress conditions, there are stress-specific, gradual changes in the transcription dynamics of genes uninvolved in stress-response pathways. The non-involvement of the probe gene in the stress-response pathways and the time necessary for the changes to occur suggest that these changes are an outcome of the global stress-response mechanisms of *E. coli*. The differences in the changes observed in the two tested stress conditions suggest that these mechanisms possess significant sensitivity.

## Materials and Methods

### Chemicals

The components of Lysogeny Broth (LB) (Tryptone, Yeast extract and NaCl) and antibiotics for *E. coli* cultures are from Sigma-Aldrich (Finland). To induce stress, 30% hydrogen peroxide (H_2_O_2_) and 4-morpholine-methanesulfonic acid (MES) were bought from Sigma-Aldrich. To perform qPCR, cells were fixed with RNAprotect bacteria reagent (Qiagen). Tris, EDTA and lysozyme for lysis buffer were purchased from Sigma-Aldrich. Total RNA extraction was done with the RNeasy RNA purification kit (Qiagen, Finland). For reverse transcription and genomic DNA removal, the Qiagen Quantitect reverse transcription kit was used. iQ SYBR Green supermix for qPCR was purchased from Biorad (Finland). Primers are from Thermoscientific and cDNA standard from qstandard. Agarose for microscopic slide gel preparation and electrophoresis, Isopropyl β-D-1-thiogalactopyranoside (IPTG) and anhydrotetracycline (aTc) for induction of cells are from Sigma-Aldrich. For staining DNA and RNA on gels, SYBR-Safe from Invitrogen (Finland) was used.

### Bacterial strain and growth conditions

We used *E. coli* strain DH5α-PRO (identical to DH5α-Z1), [46] a kind gift from Ido Golding of the University of Illinois. It contains two genetic constructs: (a) a multi copy plasmid pPROTet-K133 carrying P*_LtetO1_*-*MS2d-GFPmut3* [39], and the F plasmid based single copy pTRUEBLUE-BAC2 vector, with a P*_lac/ara-1_* promoter controlling the production of a message containing mRFP1 [70] up-stream of a 96 MS2 binding site array (P*_lac/ara-1_-mRFP1-96BS*) [55]. The *E. coli* strain DH5α-PRO, produces all necessary regulatory proteins for these constructs, namely, LacI, TetR and AraC, from the chromosome, ensuring stable tightly regulated conditions for transcription [46]. For culturing, we used LB-Lennox broth (10 g of tryptone, 5 g of yeast extract, and 5 g of NaCl per litre, of pH 7.0 with appropriate antibiotics (34 µg/ml of chloramphenicol and 35 µg/ml of Kanamycin) and incubated at 37°C with shaking.

### Induction of target gene and of the reporter gene

Cells from overnight cultures were diluted (1∶200) into fresh LB medium supplemented with antibiotics and incubated at 37°C, with shaking for exponential steady-state growth. To this culture, anhydrotetracycline (aTc) 100 ng/ml and L-arabinose (0.1%) were added first, to induce P*_LtetO-1_* for MS2d-GFP production and for pre-activating P*_lac/ara-1_* of the target gene, respectively [55]. After 1 hour, IPTG (1 mM) was supplemented to this exponential phase culture to complete full induction of P*_lac/ara-1_* [55]. Once transcribed, the target mRNA (mRFP1-96BS) is quickly tagged by MS2d-GFP proteins and can be detected as a fluorescent spot under confocal fluorescent microscope. Note that these tagged RNAs, in general and for all practical purposes of microscopy measurements 3–4 hours long, can be considered to be ‘immortal’ [52], [55].

### Stress conditions

We employed sub-lethal stress conditions to allow long live cell imaging sessions. Oxidative stress and acidic shift stress were induced by adding, respectively, 0.6 mM of H_2_O_2_ [2] and 150 mM of MES to the culture in exponential phase. Upon addition of MES, the pH very quickly shifts from 7.0 to 5.0 [12]. To confirm if stress responses were induced, we measured cell growth rates from the absorbance at OD 600 nm with a Spectrophotometer (Ultrospec 10, GE healthcare), every 30 minutes up to 4.5 hours (see File S1).

### Verification of lack of activation of the target gene by stress

To verify whether the acidic shift (pH 5.0) or oxidative stress (0.6 mM) activate the expression of our target gene by some unknown means, we acquired images of cells in stress and in optimal conditions, without inducing the target gene. Namely, cells in exponential phase were induced only for the reporter (MS2d-GFP) with aTc (100 ng/ml) for 1 h. Following this, cells were subject to stress for one hour. For control, other cells were incubated in optimal growth conditions for 1 h also. From each sample, 8 µl of cells were placed on a 1% LB agarose gel pad for microscopy imaging.

### Quantitative PCR for mean mRNA quantification

qPCR was performed to measure changes in the mean transcript rate of the target gene (mRFP1-96BS) in response to acidic shift and oxidative stress, relative to optimal growth conditions. For RNA extraction, 5 mls of cells in exponential phase was induced by L-arabinose (0.1%) and IPTG (1 mM) for 2 h at 37°C with shaking. From that, 5×108 cells were immediately fixed with RNAprotect bacteria reagent, followed by enzymatic lysis with Tris-EDTA lysozyme (15 mg/ml) buffer (pH 8.0) along with Proteinase K digestion, as per manufacturer's instruction for *E. coli* grown in LB medium. From the lysed cells, the total RNA was isolated with the RNeasy RNA purification kit. For RNA integrity assessment, 1% agarose gel electrophoresis with SYBRSafe Gel Staining was performed. The RNA was found intact with discrete bands for 16 S and 23 S ribosomal RNA. The A 260 nm/280 nm ratio of the RNA samples assessed using Nanovue Spectrophotometer (GE Healthcare) were 2.0–2.1, indicating highly purified RNA. The yield was estimated to be 0.8–0.9 µg/µl. Reverse transcription and genomic DNA removal were performed simultaneously with Qiagen Quantitect Reverse transcription kit, as per manufacturer's instructions, from 1 µg of total RNA. The synthesized cDNA was stored at −20°C.

The primers for the target mRNA (Forward: 5′AGGGCGAGATCAAGATGAGG 3′ and Reverse: 5′ GTGTAGTCCTCGTTGTGGGA 3′) were designed for a region of mRFP1 (GenBank Accession Number: AF506027) [71] with amplicon length of 154 bp. For calibration, mRFP1 DNA of 1×10^2^, 1×10^3^, 1×10^4^, 1×10^5^, and 1×10^6^ copies were mixed with the same amount of background cDNA from cells lacking the target gene (*mRFP1-96BS*) and used to generate the linear standard curve. The final reaction of 20 µl volume containing iQ SYBR Green supermix, Primers (400 nM) and cDNA template (2 ng/µl) was carried out in low-profile tube strips using MiniOpticon Real time PCR system (Biorad). The reaction protocol was 94°C for 15 s, 59°C for 30 s, and 72°C for 30 s up to 39 cycles, with fluorescence detection and melt curve analysis being performed in each reaction. No-RT controls and no-template controls were used to crosscheck non-specific signals and contamination. Finally, the Cq values were generated by the CFX ManagerTM software. The data were analysed using the Pfaffl method of normalization of gene expression [71]. We performed three independent experiments per condition, each with three replicates.

### Time-lapse microscopy

To measure the kinetics of RNA production in individual cells in optimal and stress conditions, time-lapse microscopy was performed. For this, LB media was warmed, one hour prior to the measurements in a static incubator. Cells from overnight cultures were diluted (1∶200) into fresh LB with antibiotics and are allowed to attain exponential steady-state growth. The cells were now added with aTC (100 ng/ml) to get full induction of reporter (MS2d-GFP) and L-arabinose (0.1%) to pre-activate the target gene and left in incubator for 1 hour. These exponentially growing cells (OD_600_-0.45) were now placed on a microscope slide between a coverslip and 1% LB agarose gel pad. The slide was kept in a temperature-controlled chamber (Bioptechs, FCS2) and cells were supplied with medium containing all three inducers (aTc for the reporter gene and IPTG and Arabinose for the target gene) and chemicals for stress induction, using the concentrations described above. The stress agents (oxidative stress and acidic shift inducers) were added to the warmed media, the moment preceding the start of the imaging. In the case of acidic shift, the pH of the media was found to be 5.0 at this stage and subsequently.

Throughout the measurement period, the temperature was kept at 37°C and the medium was continuously refreshed at 40 ml/hr by a micro-perfusion peristaltic pump (Bioptechs). Cells were visualized in a Nikon Eclipse (Ti-E, Nikon, Japan) inverted microscope with a C2 confocal laser scanning system using a 100× Apo TIRF (1.49 NA, oil) objective.

Images were taken with Nikon NIS-elements software. Images were acquired using a 488 nm argon ion laser (Melles-Griot) and a 515/30 nm detection filter with a 2.4 µs pixel dwell (total image acquisition time of 2.5 s). Image acquisitions began immediately after stress application, once per minute for 4 hours (under dark conditions to prevent photolytic activities). Example movie of a time series of an individual cell with contrast enhanced is shown in Movie S1.

### Image and data analysis

**Figure 4:**
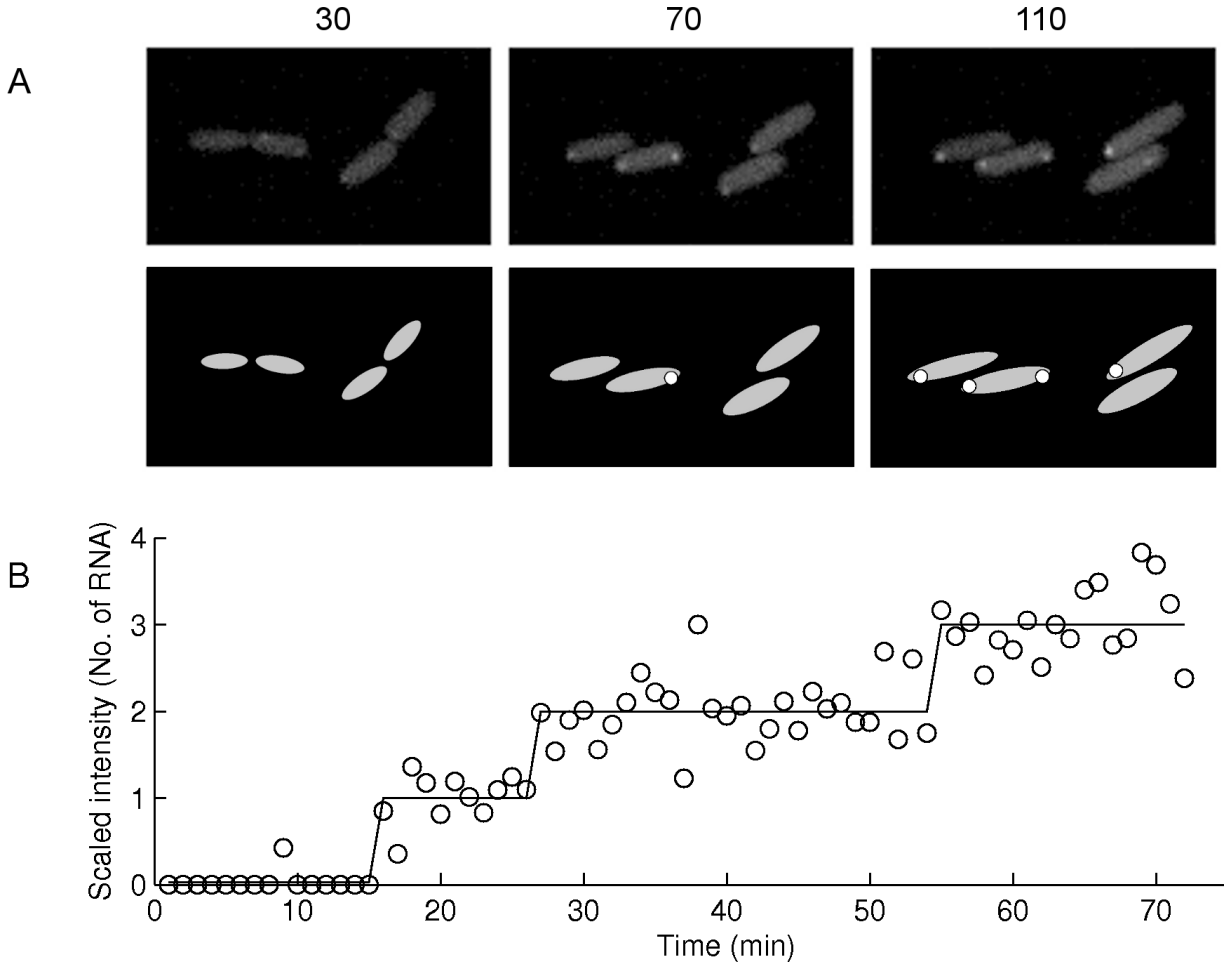
Measurements along with the results from image and data analysis. (A) Example image from the confocal microscope of cells with tagged RNA molecules (top images) and the segmentation and PCA of the top image, with segmented cells (grey) and spots (white) (bottom images). On top of these images is shown the minute when the images were taken, following the start of the measurements. (B) Time course of total intensity of the RNA spots in a single cell (circles) and monotone piecewise-constant fit (line). This figure does not correspond to the cells shown in (A).

Cells were detected from images as in [50] by a semi-automated method that includes manual cell masking and automatic spot detection. Principal component analysis (PCA) was used to obtain the dimensions and orientation of the cell inside the mask. Afterwards, fluorescent spots within were detected (Figure 4) by Gaussian kernel density estimation as in [72]. The total spot intensity in each cell, at each moment, was obtained by summing the background-corrected intensities of all spots.

Since the MS2d-GFP tagged RNA molecules do not degrade during measurements of a few hours [52], the moments of appearance of novel target RNAs in a cell were obtained as in [48], by least squares fitting a monotonically increasing piecewise-constant function to the corrected total spot intensity in a cell over time. The number of terms for the fit was selected by an F-test with P value of 0.01. Each jump corresponds to the production of one target RNA [48].

From the moments of appearance of RNA molecules, we calculated each time interval between productions of consecutive RNAs in each cell (here referred to as “Δt”). Intervals between RNAs of different cells (e.g. the last RNA of a mother cell and the first RNA of a daughter cell) were not recorded. These intervals were collected from the first two hours of the measurements, following the application of stress. For this, and to remove false correlations between the length of the intervals and the cell lifetime or the time for the first RNA to appear in a cell, we proceeded with a method similar to the one used in [51]. Namely, the intervals between consecutive RNAs in each cell were collected only for a time window of size t_c_, after the appearance of the first RNA of that interval.

By imposing a fixed value of t_c_ for all cells, the probability of appearance of the next RNA molecule during that period becomes uniform for all cells, irrespective of their division time. Here, the value of t_c_ was set to 75 minutes so as to minimize the probability mass being cut off from the distributions of intervals. We note that, to search for changes in the kinetics of RNA production over time, we recorded at which moment each interval was initiated, following the application of stress. This allows us to compare the distributions of intervals that were initiated during the first hour following the application of stress with the distributions of intervals that were initiated during the second hour.

Also, for the population images taken to check for the effects of stress on the dynamics of transcription of the target gene when uninduced, the total number of RNA molecules in the cells at a given moment in time was extracted not by the jump detection method, but from the total spot intensity distribution from all cells in an image as in [55]. For this, the first peak of the distribution is set to correspond to the intensity of a single RNA. The number of tagged RNAs in each spot can be estimated by dividing its intensity by that of the first peak. Finally, we extracted the time, t_0_, for the first target RNA to appear in each of the cells present at the start of the imaging [51], except for those few cells already containing a target RNA at the start.

### Inference of the number and duration of the sequential steps in transcription

The duration of each rate-limiting step in transcription, as well as their total number, can be obtained for each condition from the distributions of intervals between consecutive RNA productions as in [48], [52]. The inference method, for a given number of steps, assumes only that the duration of each step follows an independent exponential distribution. Given this, the most likely durations of the steps can be determined by maximum likelihood. The number of steps can then be determined by a likelihood-ratio test between pairs of models to reject a lower-degree model in favour of a higher-degree one. Finally, the goodness of fit of the preferred model can be independently evaluated by a KS test between the inferred model and the measured data.

This method does not inform on the temporal order of the inferred steps. Also, as reported in [48], [52], due to an unknown artifact, when the duration of the steps is similar, this method may favour solutions where the intermediate steps are of identical size. Increasing the number of samples used for the inference will decrease the size of the difference between the two steps below which this problem occurs. However, using models of transcription with exponentially distributed steps, it is possible to determine the smallest relative difference between two steps that our method of inference can distinguish, for given number of samples (i.e. of intervals between consecutive transcription events). For example, we verified that it reliably distinguishes the duration of each step, when these differ by ∼25% or more in duration, from sets of ∼250 intervals sampled from a model of gene expression. For smaller differences, the solution becomes biased towards identical values unless more samples are provided.

## Supporting Information

Figure S1 **Growth curves in control and under stress measured as optical density.** Curves are averaged from three independent measurements. Stress was applied at 150 min time point and error bars indicate the standard deviation.
(EPS)

File S1 **Supporting information.**
(ZIP)

Movie S1 **Example movie of a time series of an individual cell with contrast enhanced.**
(MPG)
